# Challenges Faced by Medical Trainees in Outpatient Management Education in Acute Care Hospitals: A Thematic Analysis

**DOI:** 10.7759/cureus.53800

**Published:** 2024-02-07

**Authors:** Ryuichi Ohta, Chiaki Sano

**Affiliations:** 1 Community Care, Unnan City Hospital, Unnan, JPN; 2 Community Medicine Management, Faculty of Medicine, Shimane University, Izumo, JPN

**Keywords:** reflection, legitimate peripheral participation, cognitive apprenticeship, general medicine, japan, continuity of patient care, geriatrics, clinical competence, outpatient department, medical education

## Abstract

Introduction

As societies age globally, medical education faces the challenge of adapting to the evolving healthcare needs of an aging population. This study focuses on the education of medical residents in outpatient departments in Japan, a country with a rapidly aging society. The research aims to understand the perceptions and challenges medical residents face in outpatient management, highlighting the areas for potential improvement in their educational framework.

Method

This study involved first-year medical residents at Fuchu Hospital in Osaka, using thematic analysis based on relativist ontology and constructivist epistemology. Data were collected through field notes and reflection sheets, documenting residents' interactions with patients, learning difficulties, and personal reflections. Semi-structured interviews were conducted to gain profound insights into their experiences and views on outpatient management education.

Results

Three main themes emerged from the analysis: The experience of continuity of care, the view regarding comprehensive management, and the gap between purposes and learning content. Residents expressed concerns about the limited opportunities for continuous patient care, leading to challenges in managing chronic diseases effectively. The focus on organ-specific specialties in acute care hospitals resulted in a fragmented understanding of patient care, particularly for elderly patients with multimorbidity. Furthermore, the study identified a discrepancy between the educational goals of outpatient management and the actual content delivered, highlighting the need for more observational experiences and practical guidance in outpatient settings.

Conclusion

The findings suggest a pressing need for a more structured, comprehensive, and personalized approach to outpatient management education for medical residents. As aging populations continue to grow, it is vital to equip medical professionals with the skills and knowledge to manage a wide range of patient conditions effectively. Improving the educational framework in outpatient departments can enhance patient care quality and prepare medical residents to meet the challenges of an aging society. This study contributes valuable insights into improving medical education in outpatient settings, particularly in aging societies like Japan.

## Introduction

The increasing prevalence of aging societies poses multifaceted challenges across various sectors, particularly medical education [1]. A significant area of focus is enhancing education for medical residents in outpatient departments [2]. Effective outpatient management is crucial for preventing acute exacerbations of chronic diseases and complications, thereby reducing hospital admissions [3]. Consequently, training in outpatient management is vital as healthcare needs to evolve dynamically alongside an aging population with complex, vulnerable conditions [4]. For medical residents, managing patients in outpatient settings requires mastering a diverse and intricate array of knowledge, skills, and attitudes [5]. This complexity arises from the need to navigate various medical fields.

Aging populations introduce unique complications, such as multimorbidity, polypharmacy, and the involvement of multiple healthcare providers, complicating outpatient management [6]. To manage patients effectively in these settings, comprehensive education of medical residents in outpatient care is imperative [7]. In response to these growing needs, Japan has proactively mandated education for medical residents in outpatient departments during their first two years in general hospitals [8]. Despite this, a significant gap in the literature and understanding of this education's real-world quality and effectiveness persists [8]. The perspectives of medical learners, particularly critical ones, have yet to be extensively examined.

Against this backdrop, our study primarily investigates the research question: “How do medical residents perceive the education they receive in outpatient departments?” Implementing outpatient management in Japanese medical education has been prompt, but the preparation in medical institutions may be insufficient, and the assessment of educational competencies is inadequate [9,10]. Exploring this question can shed light on the perceived challenges and potential areas for improvement in the current educational framework of outpatient management in teaching hospitals. Essentially, this research aims to bridge existing knowledge gaps and provide actionable insights to prepare medical residents for the challenges of aging societies. Therefore, this study seeks to elucidate the challenges and potential improvement areas perceived by medical residents in the education of outpatient management in general hospitals.

## Materials and methods

This study employed thematic analysis, grounded in ontology and epistemology, for research purposes. With their diverse backgrounds, prior experiences, and varied interests in medical specialties, medical trainees undergo training in general hospitals. This training aims to instill basic knowledge, skills, and attitudes essential for physicians, which are often contextual and self-directed. Our approach respected relativist ontology and constructivist epistemology to investigate issues in the education of medical residents within outpatient departments. Therefore, the thematic analysis was based on a qualitative framework [11].

Setting

Fuchu Hospital in Osaka is a prime example of a medical education setting, blending advanced healthcare with a commitment to academic excellence. Situated in a city known for its dynamism, the hospital has state-of-the-art facilities, including high-tech operating rooms and interactive learning centers. Its affiliation with local medical schools fosters a robust educational environment, facilitating knowledge exchange between experienced clinicians and medical students.

The hospital's comprehensive clinical services, covering various specialties, underscore its educational framework. This approach emphasizes a multidisciplinary methodology and patient-centric care. Cutting-edge technology, such as robotic-assisted surgery and telemedicine, significantly enhances both treatment and learning outcomes.

Fuchu Hospital also prioritizes cultural competence and international collaboration, creating a welcoming environment for diverse communities. The hospital extends its educational impact beyond its walls through extensive community outreach programs, promoting health awareness in the broader Osaka region. Thus, the institution is not merely a healthcare facility but also a hub for nurturing future medical professionals in an environment that mirrors the intricacies and compassion of modern medicine.

Participants

The study’s participants were first and second-year medical residents training at Fuchu Hospital. Their training, spanning two years, included mandatory experience in the outpatient department, focusing on managing acute and chronic care. Throughout their training in various medical departments, they intermittently worked in the outpatient department, adapting their roles to departmental needs. The hospital's education in outpatient management, primarily provided by the Department of General Medicine, commenced ten years ago.

The first researcher, RO, a specialist in family medicine, medical education, and public health, joined Fuchu Hospital as a visiting educator in outpatient management in September 2023. RO, with a background in hospital administration, conducted educational sessions two to three times a month. These sessions incorporated cognitive apprenticeship (CA), legitimate peripheral participation (LPP), and continual reflection, all critical to medical education [4,12,13]. The participants initially observed RO’s patient approach, followed by direct interactions under RO’s supervision and subsequent feedback. Each day concluded with reflective discussions to plan improvements in practice.

Data collection

Field note was used as data resources. RO observed the participants’ behaviors toward patients and took notes while teaching in the outpatient department, especially regarding the participants’ difficulties in learning outpatient management and their concrete remarks about their experiences in the learning. Reflection sheets were also used for data analysis. The participants wrote reflections on their outpatient learnings in the four categories: learning points, difficulties, emotions, and next steps. The participants wrote the reflection sheet after each RO educational session.

Semi-structured interviews with the two participants of each day were performed based on the contents of field notes and reflection sheets at the end of each day’s education. RO conducted semi-structured interviews to investigate the participants’ perspectives regarding the present outpatient management education in the hospital. The interview guide included four questions: What is the difficulty in learning outpatient management? What are the benefits of outpatient management education? How do you consider revising outpatient management education? Each interview lasted approximately 30 min and was recorded and transcribed verbatim. Eleven participants were interviewed during the study duration. 

Reflexivity

The study’s results emerged from the co-creation of knowledge by researchers and participants through their interactions. The research team, with diverse expertise in rural medical education, included members like RO, who held a master’s degree in medical education, public health, and family medicine. CS is a medical educator and professor specializing in community healthcare management and education. To mitigate biases, the team carefully discussed the findings from individual data analyses, exploring alternative interpretations during the data meaning-making process.

Data analysis

This qualitative study utilized thematic analysis for data interpretation, explicitly following Braun and Clarke's (2006) six-phase methodology [11]. Initially, RO immersed in the data, reviewing field notes and interview transcripts to form preliminary insights. The dataset was systematically coded to generate initial codes relevant to the research question.

Subsequently, RO, in collaboration with CS, organized these codes into potential themes, ensuring all relevant coded data extracts were included. The thematic framework underwent meticulous review, with themes refined through discussion. Each theme was defined and titled distinctly. RO and CS engaged in continuous dialogue to reach a consensus. The finalized themes, documented descriptively with impactful examples, aligned the analysis with the research questions and relevant literature. The study used NVivo 11 software (QSR International, Melbourne, Australia) for efficient data management.

Ethical considerations

The anonymity of participants was strictly maintained to ensure confidentiality. All participants provided written informed consent before engaging in each shared reading session. The study adhered to the Declaration of Helsinki and its subsequent amendments. The Unnan City Hospital Clinical Ethics Committee approved the study protocol (no. 20230030).

## Results

**Figure 1:**
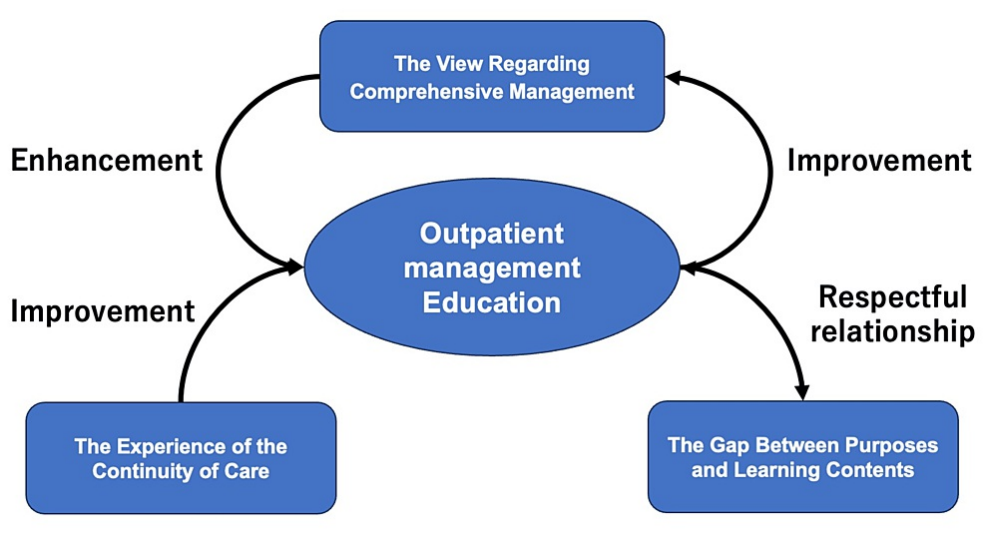
Conceptual figure of medical residents’ perceived potential improvement in outpatient management education

The thematic analysis concerning medical residents’ perceived challenges and potential areas of improvement in outpatient management education yielded insightful themes. Specifically, the study identified three themes: The experience of continuity of care, the view regarding comprehensive management, and the gap between purposes and learning content. Figure 1 illustrates the conceptual framework of this thematic analysis (Figure 1).

The experience of the continuity of care

The medical residents' experiences were predominantly limited to acute care in daily outpatient settings. This limitation in clinical settings resulted in missed opportunities for regular patient follow-up and observation of general and chronic diseases in outpatient departments. One participant explained, “My typical approach involves dealing with patients in hospitalization or emergency room situations, leaving me with fewer experiences in managing patients with mild or subacute symptoms requiring more time to resolve” (Participant 2). This situation impacted the residents’ capability to manage chronic diseases effectively through regular follow-ups. Another challenge was the lack of a fixed schedule for regular outpatient training, complicating the process of regular patient follow-up. As Participant 10 noted, “My outpatient management opportunities are irregular, making it difficult to monitor patients' conditions consistently.” Consequently, this irregular exposure led to a limited understanding of the changes in patients’ symptoms in outpatient settings, affecting their ability to make accurate prognoses or provide comprehensive advice to patients. Participant 2 further reflected, “Through reflection, I realized my limited understanding of the clinical course of diseases, which may impede my effective patient management and advice for follow-up.”

The view regarding comprehensive management

In acute care hospitals, medical residents’ experiences were often confined to interactions with organ-specific specialists, biasing their learning towards acute approaches for acute patients. This narrow focus created confusion in managing general outpatient care, especially in differentiating diagnoses and managing chronic symptoms presenting acutely. As Participant 3 remarked, “Reflecting on my clinical experiences, which were mainly in acute care across medical specialties, I find myself focusing predominantly on organ-specific differential diagnoses.” Participant 4 added, “Understanding the interplay between acute symptoms and chronic diseases in older patients is challenging due to my focus on specific organs rather than a comprehensive view.” This approach led to isolated knowledge, causing difficulties in managing elderly patients with multiple coexisting diseases. Participant 8 shared, “I struggled to understand the association between various chronic diseases, leading to confusion in managing older patients with complicated disease interactions.” Participant 2 suggested, “Learning about multimorbidity might require additional strategies beyond the usual organ-specific rotations.” Despite the limitations of learning comprehensive care in acute care settings, medical residents expressed a desire to learn more about managing patients with multimorbidity in outpatient settings. Participant 9 stated, “The current learning context has its limitations due to the hospital's role and workforce. However, I am keen to learn about managing multimorbidity in outpatient settings.”

The gap between purposes and learning contents

The absence of sufficient opportunities to observe actual outpatient consultations conducted by supervising physicians resulted in medical residents being unable to clearly understand the objectives and specific approaches required in outpatient care. This led to a lack of confidence in conducting outpatient consultations. Participant 11 expressed, “Reflecting on the learning sessions, I realize the need for more observation of general physicians and medical teachers managing outpatients. This would help me establish a clearer approach to outpatient management.” The lack of comprehensive knowledge and experience in outpatient care also contributed to an underdeveloped approach to outpatient consultations, increasing anxiety among residents. Participant 4 stated, “For the improvement of learning in outpatient management, I require more structured learning on how to approach patients in outpatient settings.” Furthermore, the medical residents' diverse future visions and motivations created a gap between their individual learning needs and the content provided in outpatient management training. Participant 6 shared, “While I may not plan to become a general physician, I am motivated to learn comprehensive patient care through observation of general physicians.” Participant 4 also expressed a desire for more hands-on experience, stating, “As someone interested in general medicine, I need more opportunities to manage patients in outpatient departments alongside general physicians.”

## Discussion

This study's thematic analysis illuminates the current state of outpatient management education for medical residents, especially in Japan's aging society. The findings uncover critical insights into the challenges and perceptions of medical residents in outpatient departments, pinpointing areas where educational frameworks could be improved.

In the challenge of continuity in patient care, medical residents voiced concerns over limited opportunities for ongoing patient care in outpatient settings. Predominantly engaged in acute care, their capacity to follow up with patients and comprehend long-term management of chronic diseases was hindered [14,15]. Continuity of care is essential for patient health and quality of life [16]. Prior literature indicates that enhanced continuity of care can improve patient mortality rates and physician and patient satisfaction levels [17]. The identified gap in outpatient management experience among medical residents underscores the necessity for a more structured and consistent outpatient training approach [16,18]. This would enable residents to participate in longitudinal patient care, which is vital for grasping chronic conditions' progression and management [19]. Implementing scheduled, guided education in outpatient management could foster learning and enhance hospital healthcare quality.

Regarding the focus on comprehensive management versus organ-specific specialization, the study reveals that acute care hospitals' emphasis on organ-specific specialties has led to a somewhat fragmented understanding of patient care among medical residents. While beneficial in specific contexts, this specialization restricts their ability to approach patient care holistically, particularly for elderly patients with multiple conditions [7,20]. Previous studies emphasize that continuous exposure to general medicine is crucial for learning comprehensive care [21]. Engaging with general medical teachers allows residents to understand the interplay of multiple organs and diseases and learn effective management strategies for complex conditions [5,22]. As aging societies grow and the demand for complex patient care increases, integrating a holistic patient care approach into the curriculum becomes imperative, emphasizing the significance of understanding the interrelations of various chronic conditions.

The study also identifies a disconnect between educational objectives and content in outpatient management. Medical residents expressed a desire for more observational experiences and practical guidance in outpatient settings, underscoring the value of experiential learning. Tailored educational methods that align with residents' interests and career aspirations are crucial [2,12,23]. CA and LPP are essential in general medicine education, supported by continuous reflection [24,25]. In CA and LPP, learners should observe and emulate their medical teachers' practices for effective patient management [26]. Since each learner has unique motivations, educational support should be scaffolded by medical teachers [26]. This study suggests that outpatient management education requires a structure that respects CA, LPP, and reflection. Establishing this framework and scaffolds makes it feasible to customize training to match residents' aspirations and provide observation and feedback opportunities from experienced general physicians in hospital education.

To enhance education in continuity of care, outpatient management programs should offer more consistent and regular experiences in outpatient settings. Structured rotations in outpatient departments could ensure that medical residents engage in continuous patient care, deepening their understanding of chronic disease management [27]. Integrating training beyond organ-specific specialties is urgent to develop comprehensive patient care skills. Modules or rotations focused on comprehensive care, particularly for elderly patients with coexisting conditions, would be invaluable in general medicine [28]. Such an approach would aid residents in grasping the complexities of multimorbidity, polypharmacy, and the intricacies of treating patients with multiple healthcare providers.

Considering medical residents' diverse career paths and interests, outpatient management training should be tailored to individual needs and future specializations [29]. This could involve elective rotations in general medicine, making the learning process more relevant and engaging for residents [30].

This study, however, has limitations. Its findings, derived from a specific sample at Fuchu Hospital in Osaka, might not widely apply to other contexts or medical education systems. The experiences and perspectives of medical residents at this single institution may not fully represent those in different regions or countries with distinct healthcare systems and training methodologies. Additionally, the study's qualitative nature may not encompass the full range of experiences and opinions among medical residents. Incorporating quantitative data or a larger sample size could have provided a broader understanding of the issues. Finally, the thematic analysis is inherently subjective, and despite rigorous efforts to ensure objectivity and consider alternative viewpoints, different researchers might interpret the data differently, leading to varied conclusions.

## Conclusions

This study underscores the necessity for a more structured, comprehensive, and personalized approach to outpatient management education for medical residents. In an era of aging populations and evolving healthcare demands, it is crucial to equip medical professionals with the skills and knowledge to effectively manage a wide array of patient conditions. Addressing the gaps identified in this study could enhance training programs, improve patient care, and better prepare medical residents for the challenges an aging society presents.
